# Temporomandibular Joint Defects: Etiology and Reconstruction Strategy: A Narrative Review

**DOI:** 10.3390/medicina62091754

**Published:** 2026-09-11

**Authors:** Hengyu Zou, Bimeng Jie, Haoliang Chen, Yang He, Shuo Chen

**Affiliations:** Beijing Key Laboratory of Digital Stomatology, National Engineering Laboratory for Digital and Material Technology of Stomatology, National Clinical Research Center for Oral Diseases, Peking University School and Hospital of Stomatology, Beijing 100081, China; 2611210500@stu.pku.edu.cn (H.Z.); jiebimeng0522@163.com (B.J.); chl0596@outlook.com (H.C.)

**Keywords:** temporomandibular joint, reconstructive surgical procedures, review

## Abstract

Temporomandibular joint (TMJ) defects primarily arise from congenital malformations, bony ankylosis, and radical tumor resection. As a pivotal structure for regulating maxillofacial development and maintaining stomatognathic homeostasis, the anatomical restoration and functional reconstruction of the TMJ are essential for rehabilitating the dentofacial complex. Distinct from standard mandibular reconstruction, TMJ reconstruction is inherently more complex, with treatment heavily dependent on etiology, age, defect extent, and coexisting dentofacial deformities—factors that invariably require patient-specific protocols. This review systematically evaluates current reconstructive paradigms, including free bone grafting (costochondral and coronoid process grafting, vertical ramus osteotomy), vascularized free flaps (vascularized fibula, vascularized medial femoral condyle, and deep circumflex iliac artery flaps), mandibular ramus distraction osteogenesis, and total joint replacement. We provide a critical analysis of their respective clinical indications, advantages, and limitations, while discussing emerging trends and future perspectives in the field.

## 1. Introduction

The TMJ is a complex bicondylar articulation comprising the mandibular condyle, glenoid fossa, articular disc, fibrous capsule, and associated ligaments. It plays an indispensable role in craniomaxillofacial development and facilitates essential physiological functions such as mastication, swallowing and pronunciation. TMJ defects, primarily involving condylar defects, can lead to mandibular movement disorder, compromised masticatory efficiency, and speech disorder [1,2]. Notably, when these defects manifest during childhood or adolescence, they often lead to secondary dentofacial deformities, severely compromising both the physiological health and psychosocial well-being of the patient.

The etiology underlying TMJ defects is multifactorial and highly heterogeneous [3,4]. Surgical interventions for congenital malformations, severe bony ankylosis, and benign or aggressive neoplasms often necessitate partial or total condylectomy, inevitably resulting in anatomical defects of varying magnitudes. Consequently, TMJ defects present with marked clinical heterogeneity, as symptoms and severity depend on the underlying pathogenesis. Due to the unique anatomical position and complicated biomechanical constraints of the TMJ, its reconstruction demands a paradigm distinct from standard mandibular reconstruction. Reconstructive algorithms must be strictly adapted, accounting for a myriad of determinants such as age of onset, concurrent dentofacial deformities, the spatial size of the defect, and the necessity for adjuvant therapies [5,6,7].

In this study, a comprehensive literature search was performed across the MEDLINE/PubMed, Web of Science, and Embase databases to retrieve relevant studies published up to July 2026. The retrieval strategy incorporated Medical Subject Headings (MeSH) and free-text terms, including: (“temporomandibular joint” OR “TMJ”) AND (“reconstruction” OR “costochondral grafting” OR “coronoid process grafting” OR “vertical ramus osteotomy” OR “fibula flap” OR “medial femoral condyle flap” OR “deep circumflex iliac artery flap” OR “distraction osteogenesis” OR “total joint replacement”). Additionally, reference lists of landmark articles and authoritative textbooks were manually screened to ensure coverage of the literature.

This review provides a systematic categorization and critical evaluation of current TMJ reconstructive strategies, discussing their respective clinical indications, advantages, limitations, perioperative complications, and long-term functional prognoses, while highlighting emerging future perspectives in the field.

## 2. Etiology of TMJ Defects

### 2.1. Tumor of the Temporomandibular Joint

Tumors of the TMJ encompass a spectrum of neoplastic and tumor-like lesions arising within the joint region. These lesions may originate from the osseous architecture, the articular disc, or the synovial membrane; alternatively, they may result from local invasion of adjacent tissues or distant metastasis [8,9]. In clinical practice, TMJ-related neoplasms are relatively rare. Because primary TMJ tumors are predominantly documented in isolated case reports and small institutional series, precise population-based epidemiological incidence rates remain unestablished in the current literature. The majority of these tumors exhibit benign or intermediate biological behavior, whereas primary malignant tumors are exceptionally rare [10,11].

Surgical resection remains the primary therapeutic modality [12]. However, it inevitably results in defects of varying anatomical regions and severities. The morphology of these postoperative defects is largely dictated by the tumor’s biological nature. Following the excision of benign condylar tumors, defects are typically localized to the condyle itself or the interpositional tissues. Conversely, the management of invasive tumors often causes complex defects involving the condyle, the articular disc, and the skull base. Such cases frequently require adjuvant postoperative radiotherapy to consolidate oncological control, further complicating the subsequent reconstructive process.

### 2.2. Temporomandibular Joint Ankylosis

TMJ ankylosis is a disabling disorder marked by long-term, worsening limited or total loss of jaw movement caused by tissue damage [13]. It is clinically categorized into intra-articular and extra-articular ankylosis. When the onset occurs during childhood or adolescence, it is frequently associated with secondary dentofacial deformities and growth disturbances.

The primary pathological hallmark of extra-articular ankylosis is scar contracture; as the internal joint architecture typically remains intact, this form does not usually result in primary osseous defects. In contrast, intra-articular ankylosis—predominantly triggered by joint trauma in pediatric and adolescent populations—is characterized by bony or fibrous adhesion, the obliteration of the joint space, and the fusion of the condyle to the articular fossa [13].

Surgical intervention represents the primary therapeutic method for TMJ ankylosis. Historically, surgical techniques have been classified into four paradigms according to range of ankylosis: lateral arthroplasty, gap arthroplasty, interposition reconstruction, and total joint reconstruction. However, contemporary clinical practice prefers classification systems based on anatomical and radiological severity rather than purely surgical techniques. The Sawhney classification (Types I–IV) remains the most widely adopted system for evaluating the extent of bone fusion, while modern high-resolution CT-based classifications (such as the He et al. scheme) have gained favor for tailoring precise reconstruction protocols [14]. These refined systems supersede older paradigms because they directly correlate pathology—such as whether the ankylosis presents as fibrous adhesion, lateral bony fusion, or complete loss of joint architecture—with specific reconstructive indications, ensuring complete resection while minimizing recurrence and preserving facial growth [14].

### 2.3. Congenital Malformations

Congenital malformations represent a critical etiological category for TMJ defects, particularly those involving the condyle. Unlike acquired defects—such as those resulting from trauma or inflammation—congenital TMJ anomalies are primary lesions arising during embryonic morphogenesis. These conditions typically necessitate complex, multi-stage treatment protocols over a prolonged clinical course [15,16]. Beyond the profound impairment of maxillofacial development, masticatory efficiency, and mandibular mobility, these malformations often exert a significant psychological burden on patients. Notably, congenital TMJ defects rarely manifest in isolation; they are predominantly phenotypic components of multisystemic syndromes, most notably first and second branchial arch syndrome (hemifacial microsomia) and Treacher Collins syndrome. Based on the specific anatomical structures involved, these malformations are categorized as follows (Table 1):

#### 2.3.1. Condylar Hypoplasia or Aplasia

As a primary growth center of the mandible, congenital failure or underdevelopment of the condyle directly results in its structural aplasia or hypoplasia [17]. While these defects may be unilateral, bilateral involvement typically leads to generalized mandibular retrognathia and micrognathia. In cases of hemifacial microsomia, computed tomography (CT) or cone-beam CT typically reveals a dysmorphic or absent condyle on the affected side, coupled with a shortened mandibular ramus, ultimately manifesting as significant mandibular deviation and facial asymmetry.

#### 2.3.2. Dysplasia of the Articular Fossa and Articular Tubercle

Dysplastic development of the articular fossa and articular tubercle often results in a flattened or absent articular surface, depriving the condyle of its physiological pivot and guided functional trajectory. For instance, in patients with cleidocranial dysplasia, imaging frequently demonstrates a shallow, curved, or completely flattened fossa alongside an underdeveloped articular tubercle [18,19]. Even in the presence of a condyle, the lack of a defined “fossa” prevents stable seating and articulation, leading to dysfunction, mandibular dyskinesia, and the potential exacerbation of micrognathia.

#### 2.3.3. Developmental Anomalies of Associated Soft Tissues and Musculature

The first and second branchial arches also give rise to the muscles of mastication and facial expression, the parotid gland, and middle ear structures. The agenesis or fibrotic degeneration of these soft tissues further compromises joint mechanics [20,21]. For example, in Nager syndrome, dysplasia or fibrosis of the masticatory and facial muscles is common. These muscular deficiencies exacerbate osseous dysplasia of the condyle and fossa, serving as a primary driver for severe restricted mouth opening and global mandibular dysfunction [22,23].

## 3. Goals and Influencing Factors of TMJ Reconstruction

The TMJ is the only movable joint in the cranio-maxillofacial region [13]. By articulating the mandible with the cranial base, it facilitates essential physiological functions, including mastication, swallowing, and articulation. Consequently, the TMJ serves as a cornerstone for maintaining the homeostatic equilibrium of the stomatognathic system. Severe structural disintegration of the joint—whether precipitated by pathological trauma, neoplastic invasion, or congenital anomalies—directly results in global stomatognathic dysfunction [10,12,13,17]. Such impairments severely compromise a patient’s fundamental activities of daily living, particularly nutritional intake and verbal communication, thereby profoundly diminishing their quality of life. Thus, TMJ reconstruction is not only an indispensable component of clinical maxillofacial rehabilitation but also remains a primary focus of advanced research in oral and maxillofacial surgery.

### 3.1. Goals of TMJ Reconstruction

The objective of TMJ reconstruction is to surgically restore both the anatomical integrity and function of the damaged joint, thereby optimizing the patient’s stomatognathic function, facial esthetics, and overall well-being [24]. The specific therapeutic goals are categorized into three primary domains:

#### 3.1.1. Anatomical Reconstruction

The primary aim is to re-establish the osseous continuity and structural integrity of the joint, encompassing the condylar, the articular fossa and the articular disc. By restoring the physiological morphology to a near-normal state, the reconstruction ensures the restoration of the joint’s intrinsic biomechanical properties and load-bearing capacity [25].

#### 3.1.2. Functional Rehabilitation

The intervention seeks to restore essential physiological processes, including mastication, deglutition, and phonation, while achieving stable occlusion and a functional range of mandibular opening. Furthermore, the reconstruction aims to eliminate symptoms such as arthralgia, crepitus, and trismus, ensuring coordinated and fluid mandibular movement [26]. A critical focus is placed on enhancing joint mobility and stability to prevent postoperative ankylosis or persistent dysfunction.

#### 3.1.3. Aesthetic Rehabilitation

Surgical reconstruction aims to correct dentofacial deformities—such as facial asymmetry or localized soft/hard tissue collapse—resulting from the initial defect or prior surgical interventions. By re-establishing harmonious jaw and joint morphology, the procedure significantly improves facial symmetry and restores the patient’s psychological confidence regarding their physical appearance [27].

### 3.2. Influencing Factors of TMJ Defect Reconstruction Strategies

Influencing factors of TMJ defect reconstruction strategies include age, defect range, facial deformity, occlusal function, tumor nature, and others. Therefore, in most cases, it is necessary to develop individualized reconstruction strategies based on the patient’s specific conditions.

#### 3.2.1. Age and Growth Potential

As a primary growth center of the mandible, the structural and functional integrity of the condyle directly modulates mandibular morphogenesis. Consequently, patient age serves as a pivotal determinant in framing the therapeutic algorithm and evaluating long-term prognoses [28,29]. In pediatric populations undergoing growth and development, the primary goal of TMJ reconstruction is to preserve and foster the intrinsic growth potential of the mandible. This early intervention aims to reduce secondary dentofacial deformities and promote harmonious skeletal and soft-tissue development. Conversely, in skeletally mature adult patients, the therapeutic paradigm shifts toward the high-precision restoration of physiological function and the concurrent correction of pre-existing dentofacial deformities, thereby optimizing stomatognathic function, facial esthetics, and overall quality of life [6].

#### 3.2.2. Anatomical Extent of the Defect

The individualization of surgical boundaries and reconstructive design must be precisely mapped to the spatial configuration of the lesion, requiring a comprehensive assessment of the condyle, articular fossa, articular disc, and adjacent soft-tissue architectures. When the defect is strictly localized to the condylar and the articular disc remains structurally and functionally intact, clinicians possess greater therapeutic flexibility; the selection of the reconstructive modality can be dynamically adapted to the patient’s age, functional demands, and institutional resources. However, when extensive disease necessitates the ablation of contiguous structures—creating a composite defect involving the condyle, interpositional materials, and infratemporal musculature—the utilization of chimeric or composite free tissue flaps becomes mandatory to achieve synchronized anatomical volumetric restoration and functional rehabilitation [30,31].

#### 3.2.3. Concomitant Dentofacial Deformities and Occlusal Stability

Due to the unique anatomical position of the TMJ, pathologies such as tumors, ankylosis, and congenital malformations are frequently coupled with severe dentofacial discrepancies. Driven by recent innovations in digital workflows and biomaterials, the contemporary standard of care has shifted from a multi-stage surgical model to a single-stage surgical approach. Currently, most of these patients can undergo TMJ reconstruction surgery simultaneously, combined with maxillofacial orthognathic surgical procedures such as maxillary Lefort I type osteotomy and sagittal split ramus osteotomy, to simultaneously correct dental and facial deformities. Health-economic and clinical outcome studies demonstrate that this single-stage strategy substantially reduces overall treatment costs compared to traditional staged protocols. This cost-efficiency is primarily driven by a 30–45% reduction in total hospital length of stay, the elimination of secondary general anesthesia and operating room fees, and lower rates of secondary revision procedures resulting from progressive occlusal relapse [6,32,33].

Chen et al. propose that the surgical approach for condylar osteochondroma must be strictly tailored through individualized reconstruction algorithms, fundamentally based on radiographic characteristics (such as the spatial extent of tumor invasion) and the severity of concurrent dentofacial deformities. For patients presenting with complex malocclusions, a comprehensive preoperative evaluation combined with an interdisciplinary orthognathic-orthodontic sequential treatment protocol is of paramount necessity [34].

Crucially, when executing simultaneous joint reconstruction and orthognathic correction, a rigorous evaluation of the patient’s dentition and maxillomandibular relationship is imperative. Stable occlusion and dynamic joint function exhibit a bidirectional, synergistic relationship. In cases presented with dental arch discrepancies or severe malocclusion, pre-surgical orthodontics or comprehensive occlusal splint therapy should be judiciously implemented to establish a stable foundation for intraoperative maxillomandibular positioning and to secure long-term reconstructive longevity [35].

#### 3.2.4. Biological Behavior of Neoplasms

The histopathological nature of TMJ tumors directly dictates the extent of surgical resection and the necessity for adjuvant therapies. The vast majority of TMJ tumors are benign lesions characterized by slow growth and a lack of metastatic potential. For these lesions, the primary surgical objective is to maximize the preservation of joint physiology and satisfy the patient’s functional gnathic demands, provided that complete encapsulation/resection is achieved to minimize recurrence risks [36]. Concurrently, if secondary dentofacial deformities are present, immediate single-stage corrective interventions are indicated to re-establish maxillofacial harmony.

Conversely, for primary or secondary malignant TMJ malignancies, the clinical priority strictly converges on radical, oncologically clear margins and regional control to ensure long-term survival. In patients requiring post-resection adjuvant radiotherapy, the deployment of vascularized free bone flaps represents the optimal choice. Comparative clinical evaluations demonstrate that in irradiated fields, vascularized bone flaps achieve significantly superior primary osseous healing rates (91–96%) compared to non-vascularized autogenous grafts, where bone union rates drop precipitously to ~50% due to radiation-induced microvascular damage and hypovascularity [37]. Furthermore, the robust, autonomous blood supply provided by vascularized flaps offers excellent resilience against radiation damage, significantly mitigating the risks of osteoradionecrosis, tissue breakdown, and hardware exposure, thereby safeguarding long-term construct stability [37,38,39].

## 4. Reconstruction Methods of TMJ

**Figure 1:**
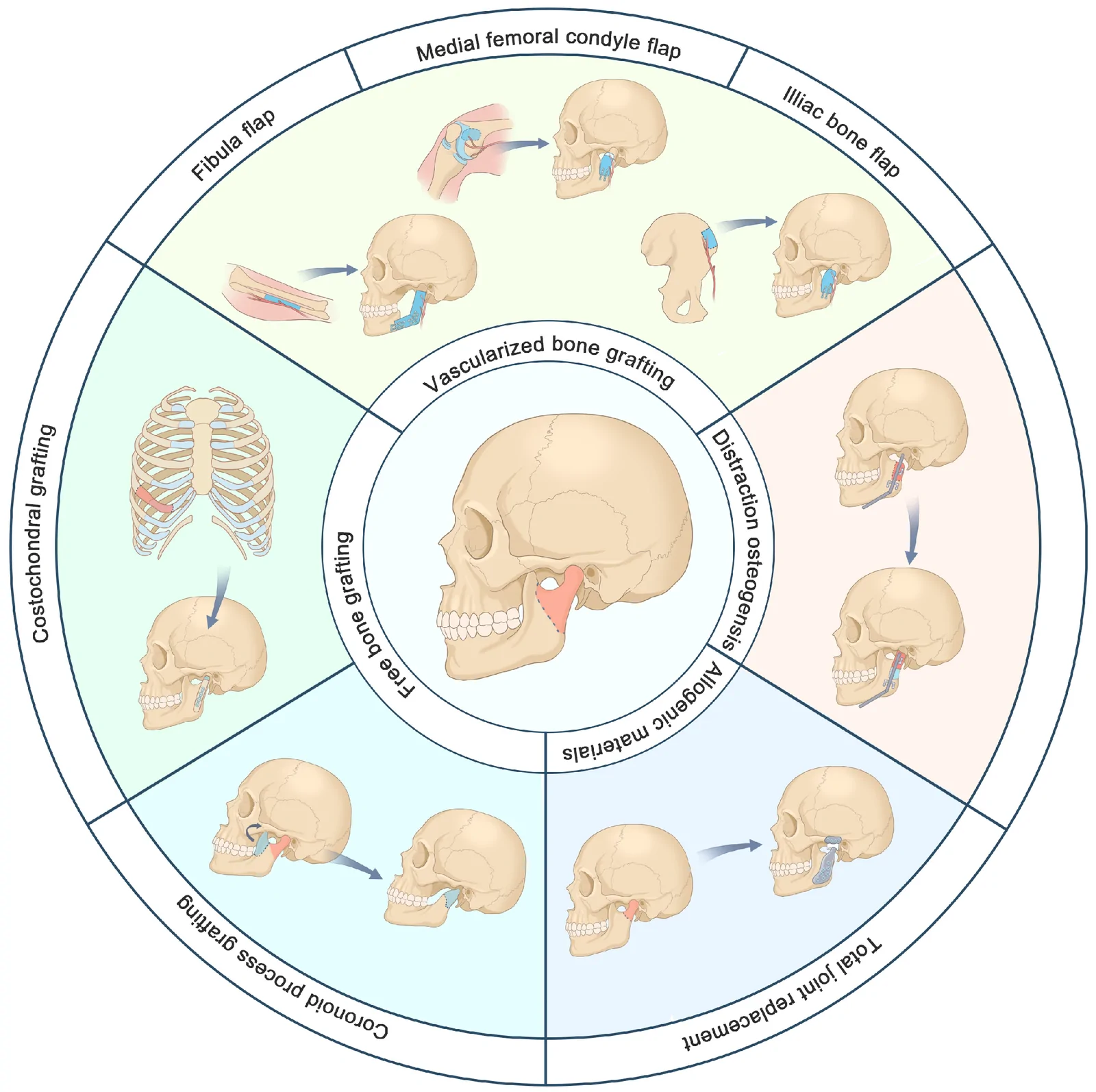
Reconstruction Strategies for TMJ Defects.

As the core component of the TMJ, the condyle plays a key role in mastication, speech, mandibular movement and facial morphology, and its reconstruction has always been a difficulty in oral and maxillofacial surgery. The goal of reconstruction is not only to restore anatomical structure, but also to maximize joint function and facial aesthetics. At present, there are many methods for the repair and reconstruction of condylar-ascending ramus defects, and reasonable repair methods should be selected according to specific conditions, including free bone grafting, vascularized bone grafting, distraction osteogenesis, total joint replacement, etc. (Figure 1).

### 4.1. Free Bone Grafting

#### 4.1.1. Costochondral Grafting (CCG)

CCG is a specialized autologous tissue transplantation procedure. The core technique involves harvesting autologous costal cartilage, which is then meticulously sculpted and transplanted to the defect site for structural or functional reconstruction. Due to the autogenous nature of the graft, this approach offers superior biocompatibility and eliminates the risk of immunological rejection. Consequently, it is widely utilized across plastic surgery, otolaryngology, and orthopedics, with the sixth rib being the most frequently utilized donor site [40].

In pediatric maxillofacial surgery, CCG is considered a mainstay for TMJ reconstruction. Its primary advantages include excellent biological integration and a growth potential that mimics the mandibular condylar growth center, thereby facilitating harmonious mandibular development. Furthermore, the substantial bone volume provided by the costal graft effectively restores the height of the mandibular ramus [41]. Our team’s retrospective study demonstrates that for pediatric type III TMJ ankylosis, CCG for joint reconstruction—following complete resection of the sclerotic osseous mass and combined with an interpositional graft (such as a temporalis myofascial flap)—presents an effective clinical modality with a low recurrence rate [42,43]. However, the procedure is associated with specific postoperative complications, including overgrowth, undergrowth, graft resorption, and infection.

The most significant challenge associated with CCG is the unpredictable postoperative growth trajectory of the graft. Guyuron and Lasa first established the unpredictable growth behavior of CCGs, noting potential overgrowth, undergrowth, or complete growth arrest [44]. Among pediatric patients treated for TMJ ankylosis, Medra found that grafts predominantly exhibited resorption and undergrowth, causing recurrent chin deviation and height deficiency [45]. Likewise, Allam et al. documented significant long-term growth lag in children with complex congenital deformities, often requiring secondary interventions [46]. Recently, Awal et al. confirmed in a 10-year cohort of 55 pediatric cases that despite functional improvements in mouth opening, dynamic growth disturbances (overgrowth or undergrowth) remain prevalent [47]. Collectively, these findings underscore that CCG growth unpredictability represents a universal clinical drawback across diverse pediatric populations [44,45,46,47].

Recent research by Zhang et al. corroborates that while CCG can simultaneously reconstruct the TMJ and improve mandibular morphology, its growth remains unstable [48]. Long-term retrospective evaluations and case series on CCG for TMJ reconstruction consistently document that graft overgrowth on the treated side is a prevalent complication, frequently resulting in secondary facial asymmetry and occlusal relapse during post-transplantation follow-up [49,50,51,52].

Three-dimensional (3D) volumetric analysis by Huang et al. revealed an average reduction in rib height of 2.44 ± 1.48 mm (representing 5.0% of the total ramus height) within 6 to 30 months. Resorption was primarily localized to the graft-ramus interface, whereas the interface between the graft and the glenoid fossa exhibited predominantly adaptive remodeling [53].

To mitigate the high recurrence rates associated with simple rib grafting, Yang et al. suggested that utilizing a temporalis myofascial flap to obliterate the surgical dead space can effectively prevent heterotopic ossification. Furthermore, successful CCG requires rigid internal fixation and comprehensive masseter muscle release. To optimize the intraoperative interincisal opening (target > 40 mm), ipsilateral or bilateral coronoidectomy is often performed [54].

At present, there is no conclusion on the effect of costochondral reconstruction in children due to long follow-up time and many influencing factors. Traditional treatment requires two steps: first reconstruct the joint, then correct jaw deformity. Zhao J et al. evaluated the long-term efficacy of costochondral grafting in growing children with TMJ ankylosis and mandibular deformity through CT measurement. The results showed that CCG is a reliable method for treating children in the growth stage with TMJ ankylosis and mandibular deformity, but its long-term effect still needs further research [55]. Lu C et al. proposed a method of digital occlusal splint (DOS)-assisted CCG in the treatment of pediatric unilateral TMJA and compared it with traditional occlusal splint. The study found that DOS can shorten treatment time and improve the accuracy of mandibular deviation correction [56]. Wei H et al. studied the effect of periosteum and hyaline cartilage preservation on bone formation after CCG transplantation. The study found that preserving periosteum and hyaline cartilage can promote bone fusion, remodeling and growth, but also increases surgical complexity and the risk of pneumothorax [57].

Chen et al. has integrated Computer-Aided Design (CAD) to streamline this process. By acquiring preoperative dental and occlusal data, we proposed that virtual mandible position-guided CCG combined with the functional appliance to improve facial deformity. Intraoperatively, a digital occlusal guide is utilized to facilitate the simultaneous correction of the mandibular deformity during joint reconstruction. Our clinical outcomes demonstrate that this integrated digital workflow effectively corrects mandibular deviation and restores facial symmetry with high predictability [41].

#### 4.1.2. Coronoid Process Grafting

Autogenous coronoid process grafting utilizes the mandibular coronoid process as a donor site for the functional reconstruction of condylar defects or joint ankylosis. A primary advantage of this technique is the elimination of a secondary surgical site, thereby reducing donor-site morbidity and simplifying the operative procedure. This approach offers excellent biocompatibility and favorable postoperative osseous remodeling. It is particularly indicated for condylar reconstruction following the treatment of TMJ ankylosis or the resection of benign and low-grade malignant tumors [58]. However, its application in patients with congenital condylar hypoplasia—especially in pediatric populations—must be approached with caution. In these cases, the coronoid process may lack sufficient length and morphological volume for adequate reconstruction, and its inherent growth potential seldom matches the contralateral side, rendering it a secondary option compared to CCG.

Free coronoid process grafting has demonstrated favorable clinical outcomes in restoring TMJ function, characterized by minimal surgical trauma, robust bone healing, and satisfactory functional outcomes. Nevertheless, as with all non-vascularized free bone grafts, it remains susceptible to postoperative infection and varying degrees of graft resorption. Furthermore, significant individual anatomical variation in coronoid process morphology can occasionally impede the attainment of the required reconstructive height [59].

Numerous studies have validated the clinical utility of this modality. Liu et al. evaluated 15 patients undergoing condylar arthroplasty with autologous coronoid process grafts; long-term follow-up revealed high patient satisfaction, successful osseointegration, and significant bone remodeling [60]. Similarly, Wang and Xu et al. reported successful outcomes with no recurrence in patients treated for bilateral true TMJ ankylosis using bilateral coronoid process grafts [61]. Comparative research by Zhang et al. demonstrated that postoperative maximal mouth opening (MMO), lateral excursive range, and dietary scores were significantly improved relative to preoperative values. Notably, their findings suggested that coronoid process grafting may be associated with a lower complication severity and recurrence rate compared to CCG [62]. However, Malhotra et al. emphasized that since this is a purely osseous reconstruction, the status of the articular disc is critical. In cases where the disc is non-functional or cannot be preserved, the risk of long-term joint degeneration remains a concern, which may compromise the overall reconstructive longevity [63].

#### 4.1.3. Vertical Ramus Osteotomy (VRO)

VRO is a reconstructive technique performed following the resection of TMJ tumors or ankylotic masses. The procedure involves a vertical osteotomy of the mandibular ramus, where the posterior border of the ramus is cranially repositioned into the articular fossa to serve as a neo-condyle. A critical feature of this technique is the preservation of the medial muscular and periosteal attachments, which maintains a robust blood supply to the pedicled bone segment. Consequently, this pedicled graft is highly resistant to resorption compared to free bone grafts, ensuring stable postoperative vertical joint height. From a clinical standpoint, VRO eliminates the need for a secondary donor site, thereby avoiding the morbidity associated with procedures such as CCG. The simplicity of the technique makes it particularly suitable for the immediate reconstruction of defects following the excision of benign condylar lesions (e.g., osteochondroma, osteoma), condylar hyperplasia, or ankylosis [63].

Despite its advantages, VRO may alter the morphology of the mandibular angle, potentially leading to an unnatural or asymmetric contour of the inferior mandibular border—a factor that necessitates careful preoperative counseling for patients with high aesthetic expectations [58]. Furthermore, VRO may be insufficient for cases involving severe osteoarthropathy, extensive destruction of the articular fossa, or those requiring a substantial increase in vertical dimension. In such complex scenarios, VRO must be supplemented with additional procedures or superseded by more comprehensive reconstructive modalities [64].

VRO has emerged as a safe and efficacious modality for TMJ reconstruction following condylar tumor resection. By utilizing a pedicled posterior ramus segment, surgeons can achieve simultaneous morphological and functional restoration, streamlining the patient’s recovery process. Success hinges upon precise osteotomy, meticulous sculpting of the neo-condyle, preservation of the vascular pedicle, and adherent postoperative rehabilitation.

Wang et al. evaluated the efficacy of VRO in patients with condylar osteochondroma. Following a minimum 12-month follow-up, all subjects exhibited successful recovery of occlusion, facial symmetry, and joint mobility. Radiographic assessment confirmed that the cranially displaced posterior ramus segment was stably positioned within the articular fossa, showing progressive remodeling into a smooth, oblate condylar morphology over time. This suggests that the neo-condyle undergoes adaptive functional remodeling [65]. Similarly, Liu et al. reported stable occlusal relationships and significant improvements in MMO without evidence of recurrence during the observation period, further establishing VRO as an ideal reconstructive choice for localized condylar defects [66].

### 4.2. Vascularized Bone Grafting

Vascularized bone grafting is a cornerstone in the reconstruction of complex, large-segment TMJ and maxillofacial defects involving both hard and soft tissues. By providing vital osseous tissue with an independent blood supply, this modality ensures high graft survival rates and robust resistance to infection [67]. It serves as a reliable biological scaffold for restoring facial symmetry, mandibular kinematics, and the subsequent rehabilitation of occlusal function [68].

Conventionally, the management of complex TMJ defects has relied on staged surgical protocols. However, these multi-step procedures entail prolonged treatment cycles, repeated surgical interventions, and a significant socioeconomic and psychological burden on the patient. Furthermore, staged approaches often complicate secondary orthognathic interventions, as progressive soft-tissue scarring and unpredictable osseous remodeling can impede the attainment of an ideal functional and esthetic outcome [69]. To address these challenges, a synchronous reconstructive strategy combining “vascularized bone grafting with simultaneous orthognathic surgery” can be implemented. This approach leverages high-precision digital workflows and virtual surgical planning to achieve the synchronous completion of tumor resection, complex joint reconstruction, and the correction of dentofacial deformities [70,71].

#### 4.2.1. Vascularized Fibula Flap (VFF)

The vascularized fibula flap is a sophisticated microsurgical technique involving the transplantation of a fibular segment with its pedicled vasculature to reconstruct mandibular and TMJ defects. First introduced by Hidalgo in 1989 for mandibular repair, the fibula has since become a preferred donor site due to its substantial length, robust vascularity, and superior bicortical strength, making it ideal for the management of extensive defects following malignant tumor resection [67].

A primary challenge in condylar reconstruction using the vascularized fibula flap is the potential for fibular malposition and the resultant suboptimal fossa–condyle relationship. As a complex diarthrodial joint, TMJ function is contingent upon precise condylar morphology, spatial positioning, and the integrity of the articular disc and associated ligamentous attachments [72]. Maintaining a stable and functional “fossa–condyle” interface remains a formidable task in the vascularized fibula flap reconstruction. Furthermore, the procedure is associated with significant surgical trauma and potential long-term donor-site morbidity. Postoperatively, the newly sculpted “neo-condyle” may undergo adaptive remodeling or resorption, which can compromise long-term joint stability.

The intricate 3D architecture of the mandible makes intraoperative contouring of the reconstruction plate and fibula exceptionally difficult when relying solely on clinical experience. Repeated adjustments not only prolong operative time but also risk compromising the delicate endosteal and periosteal capillary networks. Moreover, a suboptimal fit between the pre-contoured plate and the bone increases the risk of local inflammation and plate exposure [73]. In recent years, the integration of CAD/CAM, 3D printing, and Virtual Surgical Planning (VSP) has revolutionized the vascularized fibula flap reconstruction. Preoperative digital contouring, guided intraoperative osteotomy, and 3D model-based verification have significantly enhanced reconstructive precision and functional outcomes.

The vascularized fibula flap reconstruction effectively restores mandibular continuity and approximates condylar morphology. Research by Yin et al. demonstrated that patients undergoing VFF transplantation achieved superior occlusal recovery, improved facial symmetry, and more physiological mandibular kinematics compared to non-vascularized techniques [74]. Similarly, Zhang et al. emphasized that the spatial orientation and morphology of the fibular graft end are critical determinants of postoperative TMJ function. Clinically, most patients achieve a satisfactory range of motion, with some studies reporting a mean maximal incisal opening of 42.8 ± 5.7 mm without significant deviation. While masticatory efficiency is generally restored, electromyographic studies often reveal persistent asymmetry in masseter muscle activity, with diminished strength on the ipsilateral side. Furthermore, compared to condylar-preservation protocols, patients with fibular-replacement condyles often exhibit impaired lateral pterygoid muscle function, which manifests as an increased incidence of mandibular deviation and restricted lateral excursions [72].

In a single-institution preliminary series, Bai et al. evaluated a “capsule suspension technique,” wherein the articular capsule and disc were horizontally mattress-sutured to the neocondyle of the fibula using non-absorbable sutures [75]. Their preliminary findings indicated that this approach helped maintain a favorable anatomical relationship between the neocondyle and the articular disc, optimizing early postoperative mandibular kinematics. In their preliminary comparison with a non-suspension cohort, 7 out of 9 patients in the suspension group recovered symmetrical jaw opening trajectories, whereas the control group exhibited consistent deviation toward the affected side. While these single-center observations are encouraging, broader multi-center prospective validation is necessary to confirm long-term reproducibility and clinical superiority [75].

#### 4.2.2. Vascularized Medial Femoral Condyle Flap

The vascularized medial femoral condyle (MFC) flap is a sophisticated composite free flap comprising osseous tissue, articular cartilage, and periosteum. It is harvested with the descending genicular artery (DGA) or the superior medial geniculate artery as its vascular pedicle. Utilizing microsurgical anastomotic techniques, this flap is transplanted to the TMJ region to reconstruct the condylar, the articular fossa, or the entire joint complex.

The MFC flap represents a cutting-edge paradigm in TMJ reconstruction [76]. Its most distinctive advantage over other vascularized bone flaps (such as the fibula or iliac crest) lies in its capacity for osteochondral integration. By providing a vascularized, hyaline-like articular cartilage surface, the MFC flap facilitates the restoration of physiological joint morphology and biomechanics, approximating the goal of “true functional reconstruction.” Despite these advantages, the technique carries a high threshold for surgical expertise. Venous congestion (poor venous return) remains one of the most critical and frequent early postoperative complications. Furthermore, the inherent technical complexity and significant socioeconomic burden preclude its use as a universal first-line treatment for all TMJ defects.

The reconstructive potential of the MFC flap was highlighted in 2014 by Thiele et al., who emphasized its ability to provide a multi-component blood supply (bone, cartilage, and skin) with minimal donor-site morbidity [77]. That same year, Lee et al. reported the successful application of an MFC free flap to treat a condylar fracture malunion where the articular cartilage was absent [78]. In 2021, we further refined this technique for condylar reconstruction. In a cohort of four patients, we reported no significant intraoperative or postoperative complications, such as infection, hemorrhage, or facial nerve paresis. Postoperative computed tomography (CT) and 3D volumetric analysis demonstrated successful graft integration and excellent anatomical fidelity. While mandibular height and facial symmetry were effectively restored, it was noted that the bulk of the transplanted flap could result in residual soft-tissue fullness on the affected side [76].

Chen et al. investigated three distinct fixation configurations—overlapping, beveled, and flush techniques—for TMJ reconstruction utilizing a medial femoral condyle flap. The study employed the Finite Element Analysis method and demonstrated that all three fixation configurations successfully satisfied the biomechanical requirements for TMJ reconstruction, presenting no significant risk of plate or screw fracture, or pronounced displacement of the MFC bone graft. Notably, the overlapping configuration exhibited the minimum displacement and stress distribution. Conversely, while the flush configuration generated the maximum stress concentration on the plates and screws, it imposed the least stress on the cancellous core of the MFC bone graft, and the flush technique requires the minimum bone graft volume and yields a more esthetically favorable postoperative facial contour [79].

As for further study, we evaluated the reconstructive outcomes of a vascularized MFC flap in a unilateral TMJ model using five adult male miniature pigs, with the unoperated contralateral side serving as the control. Postoperative radiographic assessments and histological evaluations demonstrated that the vascularized MFC osteochondral flap not only restored the mandibular deficiency, but also seamlessly integrated into the functional system of the TMJ at the histological level. This integration was achieved through an adaptive morphological evolution characterized by the “Fibrosis of hyaline cartilage” effectively overcoming the critical drawbacks of conventional autogenous cartilage grafting. These findings indicate that the MFC flap possesses substantial potential as a viable alternative modality for TMJ reconstruction [80].

#### 4.2.3. Deep Circumflex Iliac Artery Flap (DCIA)

The vascularized iliac crest flap, specifically the deep circumflex iliac artery (DCIA) flap, is a versatile composite free flap. It incorporates the iliac bone along with associated musculocutaneous components, pedicled on the deep circumflex iliac vessels or their perforators. Due to its substantial osseous volume, robust blood supply, and a natural curvature that closely approximates the mandibular anatomy, DCIA has become a premier choice for reconstructing large-segment mandibular defects involving the condylar unit [81,82].

The iliac crest provides a generous amount of naturally contoured bone, whose arcuation is highly congruent with the mandibular angle and inferior border. This inherent morphology facilitates anatomical shaping and restores mandibular continuity with high fidelity [81,83]. However, when the distal aspect of the iliac bone is utilized for condylar reconstruction, its morphology differs significantly from the physiological condyle. This discrepancy can lead to suboptimal seating within the articular fossa or poor adaptation to the articular disc, potentially resulting in functional outcomes inferior to other specialized modalities. Furthermore, the necessity of a secondary surgical site and the perceived magnitude of donor-site morbidity have historically limited its application. Nevertheless, advances in digital surgical planning and microsurgical techniques have enabled DCIA to effectively restore facial esthetics, occlusal stability, and joint kinematics, particularly in complex cases involving contiguous defects of the condyle, ramus, and mandibular body.

The study by Gao et al. demonstrated that the vascularized deep circumflex iliac artery flap was significantly superior to the fibular flap in restoring the vertical height of the mandible. Consequently, the iliac crest flap provides a more robust bone volume foundation and facilitates superior recovery of occlusal relationships during subsequent dental implant placement and functional occlusal reconstruction. At the 24-month postoperative long-term follow-up, marked improvements were observed in both the patients’ quality of life and MMO. For cases where the defect is localized to the mandibular angle and ramus, and the primary load-bearing surface of the condyle remains intact, the iliac crest flap can better simulate the anatomical curvature of the mandibular angle, thereby yielding postoperative joint movement trajectories that align more closely with physiological characteristics [84].

Lin et al. found that titanium miniplates function as a “load-sharing” fixation system, which permits the bone graft to bear moderate physiological stress, thereby stimulating osseous healing and bone remodeling. Consequently, during mandibular reconstruction utilizing a DCIA flap that encompasses the joint–ramus region, the prioritized use of 2.0 mm titanium miniplates for multi-point fixation is recommended, provided that the residual bone quality and quantity are adequate, to minimize long-term complications [85].

The literature indicates that the survival rate of the DCIA exceeds 95% [86,87]. Long-term outcomes are characterized by stable osseous remodeling and a remarkably low incidence of secondary ankylosis or significant resorption [88]. By simultaneously reconstructing the masticatory muscle attachments and associated ligamentous tissues, this integrated approach moves closer to achieving comprehensive anatomical and functional rehabilitation.

#### 4.2.4. Pediatric Composite Defects

The research conducted by Zhang et al. revealed that the mandible is the most common site of jaw tumors (accounting for 22.15% of all pediatric lesions), with resections frequently resulting in extensive structural and functional deficits [89]. Reconstruction of composite mandibular defects in pediatric patients—especially those involving combined osseous, condylar, and extensive soft-tissue deficits following ablation of malignant or aggressive tumors—presents a unique surgical challenge. Unlike adults, treatment algorithms in children must reconcile immediate structural/soft-tissue coverage with long-term craniomaxillofacial growth. In these composite defect beds, non-vascularized bone grafts or isolated costochondral grafts (CCGs) often prove inadequate due to unpredictable resorption and a lack of a vascularized soft-tissue paddle.

Zhang et al. proposed VFFs serve as a primary workhorse option, providing both structural stability and soft-tissue coverage essential for primary healing [89]. A primary long-term concern regarding pediatric VFF reconstruction is the potential growth mismatch between the non-epiphyseal graft and the surrounding craniomaxillofacial skeleton, which often necessitates multi-stage secondary interventions. In pediatric patients presenting with complex composite defects, vascularized bone flaps address immediate structural losses but present distinct secondary challenges.

Some longitudinal quantitative evaluations reveal that pediatric free fibular grafts undergo active physiological adaptation under functional loading rather than behaving as static bone blocks [90,91]. The long-term 3D-CT morphological tracking conducted by Liu et al. indicates that when the proximal fibula is positioned in the glenoid fossa, it can undergo biomechanical remodeling to form a functional neocondyle. Furthermore, progressive axial lengthening is observed in both the reconstructed ramus and the residual native mandible, preserving overall facial profile symmetry into skeletal maturity (>18 years) [90]. Objective 3D cephalometric analysis confirms that pediatric mandibular reconstruction with free fibula flaps, when coupled with early functional and occlusal rehabilitation, effectively supports symmetrical midfacial development without precipitating severe secondary midfacial deformities over longitudinal follow-up [91].

Collectively, these findings demonstrate that VFF provides a reliable primary osteocutaneous substrate for pediatric composite defects. Any minor residual growth discrepancies near skeletal maturity can be definitively refined through staged secondary interventions—such as distraction osteogenesis or orthognathic surgery—to ensure optimal long-term functional and aesthetic outcomes.

### 4.3. Mandibular Ramus Distraction Osteogenesis

Mandibular ramus distraction osteogenesis (MRDO) is a regenerative technique that utilizes gradual mechanical traction to facilitate endogenous bone formation. Governed by the tension-stress principle, the application of continuous and stable distraction forces stimulates the metabolic activity of local tissues, thereby inducing bone and soft-tissue regeneration. This modality is particularly indicated for TMJ reconstruction in patients with bony ankylosis, a shortened or absent condylar–ramus unit, or defects following tumor resection [92].

Following the excision of the pathological condyle, an internal distractor is utilized to incrementally restore ramus height and reconstruct joint function. This gradual expansion not only improves MMO and facial symmetry but also enhances the overall facial profile and projection. During the consolidation phase, the superior aspect of the distracted segment—the neo-condyle—undergoes adaptive remodeling, resulting in a smooth, corticalized articular surface. Concurrently, the mechanical stimulation induces the formation of organized fibrous tissue between the bone segment and the glenoid fossa, which functions as a pseudo-disc, providing a biological interpositional layer [93].

As a “bone-transport” modality, MRDO offers the unique advantage of de novo condylar reconstruction without the need for autogenous bone grafting. While MRDO effectively increases mandibular range of motion, the therapeutic process is characterized by a prolonged treatment cycle and necessitates a secondary surgical intervention for distractor removal. We found that potential long-term complications include partial graft resorption and the development of an anterior open bite due to unpredictable vector control [94]. In recent years, biodegradable screw fixation systems have been explored in animal studies, providing critical biomechanical evidence for the development of distraction osteogenesis devices that eliminate the need for a second surgical procedure [95].

### 4.4. Alloplastic Total Joint Replacement

Alloplastic total joint replacement (TJR) involves the surgical substitution of a pathological or damaged TMJ with a prosthetic system to restore functional kinematics, alleviate chronic pain, and improve masticatory efficiency. These prosthetic systems typically consist of an articular fossa component (often ultra-high-molecular-weight polyethylene, UHMWPE) and a condylar component (typically cobalt–chromium or titanium alloy). The interplay between these materials determines the implant’s wear resistance and long-term biocompatibility [96,97].

Currently, two prominent systems dominate the clinical landscape: TMJ Concepts and Zimmer Biomet [97]. TMJ Concepts offers a patient-specific prosthesis, where both the fossa and condylar components are manufactured using CAD/CAM technology to match the patient’s unique skeletal morphology, ensuring superior fit and stability [98]. It is widely implemented in clinical practice and exhibits excellent biocompatibility. Although Zimmer Biomet manufactures custom systems in select global markets, the Biomet total joint prostheses (Zimmer Biomet, Jacksonville, FL, USA) currently approved and accessible for clinical practice in China consist primarily of standardized stock components. The standardized prostheses may not perfectly adapt to the mandibular ramus or zygomatic arch, often necessitating preoperative digital simulations and intraoperative osseous re-contouring to achieve an acceptable fit. Research into domestically developed personalized or standardized TJR systems is currently in its nascent stages, and their long-term clinical performance remains under active investigation [99].

TJR offers significant clinical advantages, including minimal donor-site morbidity (no bone harvesting required), reduced operative time, and a remarkably low recurrence rate—particularly in cases of end-stage joint disease. However, inherent biomechanical limitations persist. Most contemporary prostheses primarily facilitate translation-like sliding but lack the complex rotational dynamics of a biological joint, typically resulting in an MMO of approximately 35 mm. Furthermore, the high financial cost associated with customized TJR remains a barrier to its universal clinical adoption [100].

Driven by advancements in digital technology, VSP enables a comprehensive evaluation of critical parameters. These include the optimal fixation positioning of the alloplastic prosthesis, its adaptation to the surfaces of the zygomatic arch and mandible, potential kinetic interference from the coronoid process during mandibular movement, and associated alterations in occlusal relationships. Furthermore, VSP facilitates the simulation and planning of surgical strategies—specifically regarding the extent of intraoperative bone contouring and coronoidotomy or coronoidectomy—while allowing for the fabrication of custom surgical guides to streamline surgical maneuvers [99]. For patients with TMJ diseases accompanied by secondary maxillomandibular deformities, such as a reduced mandibular ramus height resulting from idiopathic condylar resorption, conventional sagittal split ramus osteotomy is insufficient to restore the structural ramus height. In such scenarios, TJR can simultaneously reconstruct the joint morphology and successfully restore the physiological ramus height [101,102].

For patients presenting with concomitant dentofacial deformities, the integration of simultaneous orthognathic surgery with TJR has emerged as a transformative strategy. This “single-stage” approach facilitates the synchronous correction of skeletal discrepancies and joint pathology, ensuring harmonious occlusal and functional rehabilitation. There is extensive support in the literature for the efficacy of this integrated protocol. Huang et al. highlighted that simultaneous surgery offers higher therapeutic efficiency, more stable long-term outcomes, and a reduced socioeconomic burden by consolidating multiple procedures [103]. Gomez et al. demonstrated significant improvements in pain scores, MMO, and lateral excursions, alongside highly predictable facial esthetic enhancements [104]. The study by Cui et al. investigated a transoral approach for condylectomy performed simultaneously with orthognathic surgery and demonstrated that following this therapeutic intervention, the joint spaces of the patients gradually restored to physiological ranges, and the structural integrity of the temporomandibular joint progressively stabilized [105]. Furthermore, a systematic review by Zhao et al. suggested that the combination of autologous or alloplastic joint reconstruction with simultaneous orthognathic surgery represents a widely advocated approach for managing complex TMJ ankylosis or end-stage osteoarthropathy associated with maxillofacial deformities, although the available supporting literature primarily consists of retrospective observational cohorts [24].

Long-term safety is well-documented. In a systematic review of 320 patients, Lima et al. reported an overall prosthetic survival rate of 97% over follow-up periods ranging up to 21 years. While rare, failures typically occurred within the first six months due to peri-implant infection. These data underscore TJR as a robust, safe, and highly effective modality for comprehensive TMJ reconstruction [106].

**Figure 2:**
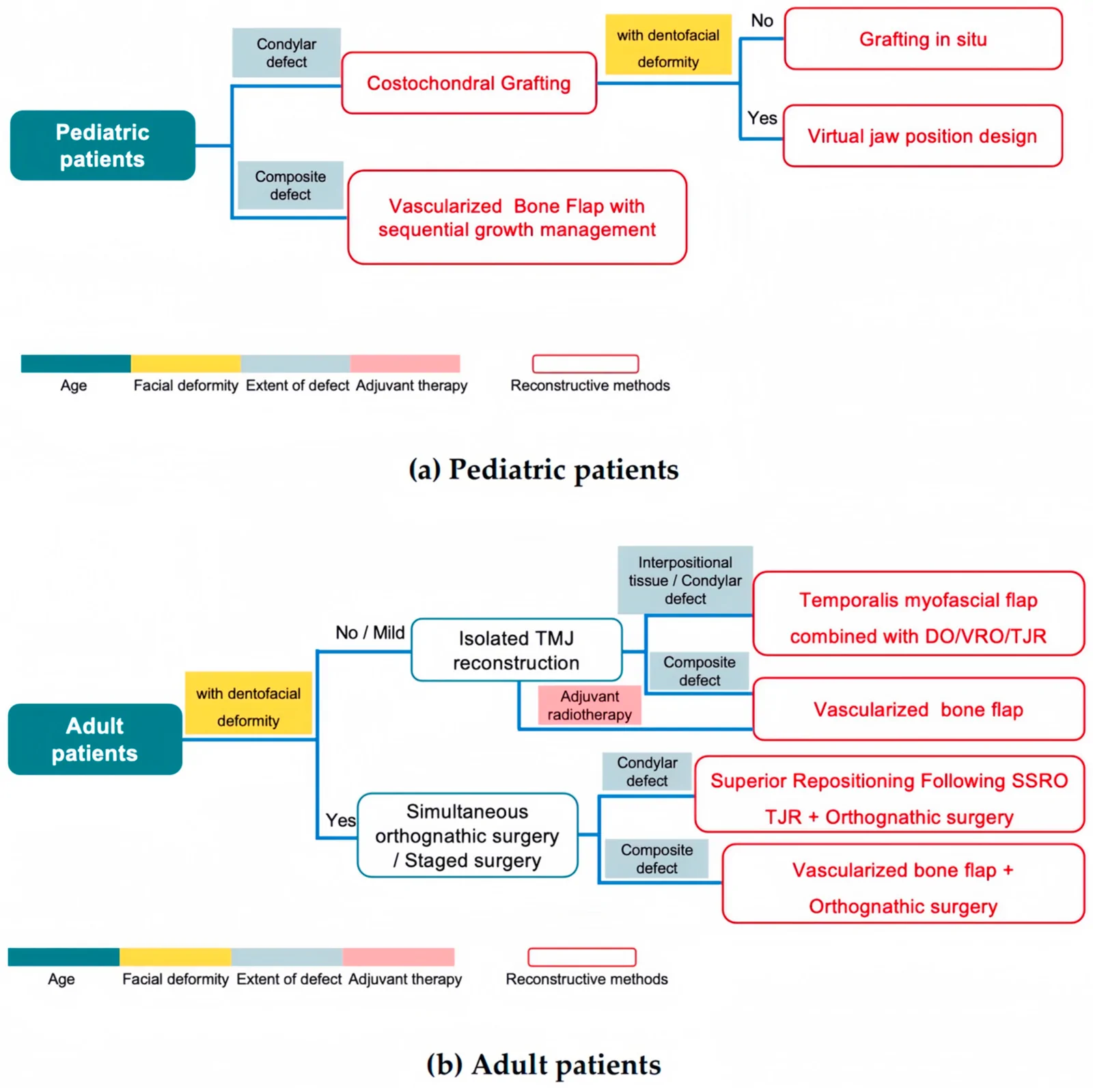
Comprehensive clinical decision-making matrix for TMJ reconstruction: (**a**) Pediatric patients: Strategic reconstructive pathway for pediatric joint defects. For isolated condylar defects, costochondral grafting (CCG) is indicated, with virtual jaw position design utilized in the presence of dentofacial deformity. For pediatric composite defects, vascularized bone flap transplantation integrated with long-term sequential growth management is recommended. (**b**) Adult patients: Stratified by dentofacial deformity, defect extent, and adjuvant radiotherapy history. In isolated TMJ reconstruction without severe deformity, options range from temporalis myofascial flaps (combined with DO, VRO, or TJR) to vascularized bone flaps depending on tissue loss and history of adjuvant radiotherapy. For cases presenting with pronounced dentofacial deformity, simultaneous or staged orthognathic surgery is performed alongside TJR or vascularized bone grafting. Abbreviations: CCG, costochondral graft; DO, distraction osteogenesis; SSRO, sagittal split ramus osteotomy; TMJ, temporomandibular joint; VRO, vertical ramus osteotomy; TJR, total joint reconstruction.

Collectively, reconstruction for TMJ defects necessitates a comprehensive evaluation of multiple factors, including the patient’s age, the presence and severity of concomitant deformities, the extent of post-oncological resection, and the requirement for adjuvant postoperative radiotherapy (Figure 2).

## 5. Challenges and Future Development Directions

### 5.1. Current Major Challenges

Based on the aforementioned modalities and current literature, TMJ reconstruction continues to face several formidable challenges:

#### 5.1.1. Limitations in Biomaterials and Prosthetic Design

While alloplastic TJR significantly improves functional outcomes, long-term concerns regarding prosthetic loosening, fatigue failure, and occlusal shifts secondary to progressive bone remodeling persist [107]. A major concern is the generation of wear debris from traditional or suboptimal materials, which can trigger localized inflammatory cascades, foreign body giant cell reactions, and periprosthetic osteolysis. Although contemporary materials have undergone rigorous refinement, the risks of mechanical fatigue, electrochemical corrosion, and tribological wear remain. Furthermore, the high degree of anatomical variability in the TMJ complex limits the efficacy of standardized prostheses, which may result in suboptimal biomechanics. While patient-specific implants offer superior anatomical adaptation, their extremely high cost and long production time remain major obstacles to broad clinical use [99].

#### 5.1.2. Deficiencies in Soft Tissue and Disc Reconstruction

The TMJ is situated within a dense network of critical neurovascular structures. Consequently, surgical interventions carry an inherent risk of complications, including facial nerve paresis, hemorrhage, and infection [108,109]. The surgical complexity and associated risks are substantially magnified in cases involving malignant neoplasms or severe, recalcitrant bony ankylosis, where anatomical landmarks may be obliterated.

#### 5.1.3. Intraoperative Complexity and Surgical Risk

A critical gap remains in the availability of an ideal articular disc replacement. Current autogenous tissue grafts are susceptible to resorption, morphological distortion, and donor-site morbidity. While tissue-engineered articular discs represent a promising frontier, they remain largely confined to laboratory settings or small-scale clinical trials and are not yet viable for large-scale application [110]. Furthermore, complex repair of the joint capsule and ligament structures—key to joint stability and smooth jaw movement—is hard to achieve [111]. Consequently, a subset of patients may continue to experience suboptimal range of motion, persistent arthralgia, or even secondary ankylosis despite technically successful osseous reconstruction [112].

### 5.2. Development Trends

Advances in tissue engineering, 3D bioprinting, and stem cell therapeutics are redefining the boundaries of TMJ reconstruction, shifting the clinical objective from “passive anatomical repair” toward “active functional regeneration.”

#### 5.2.1. Biomimetic Materials and Advanced Prosthetics

To overcome the limitations of current metal and polymer prostheses—specifically regarding wear and immunological rejection—the development of next-generation biomimetic materials is imperative. Future research focuses on novel polymers and self-lubricating composites that exhibit superior wear resistance, low-friction coefficients, and high biocompatibility [113]. Furthermore, the rise of 3D bioprinting represents a transformative leap. By precisely layering bio-inks (comprising biomaterials, viable cells, and growth factors) according to CAD models, this technology can fabricate patient-specific, biologically functional scaffolds for articular discs and condyles [114,115,116]. High-precision, multi-material co-printing remains a critical research frontier for achieving complex tissue integration.

#### 5.2.2. Digitalization and Intelligent Surgical Systems

##### Virtual Surgical Planning and AI Integration

VSP has already established itself as a standard auxiliary tool for complex TMJ reconstruction. The future will see deeper integration of Artificial Intelligence algorithms, which will automate complex 3D morphometric measurements, perform collision detection, and recommend optimal surgical trajectories, thereby enhancing planning efficiency and accuracy [117].

##### Advanced Intraoperative Guidance

Surgical navigation systems are increasingly being coupled with Augmented Reality. This synergy allows for the superimposition of critical neurovascular and skeletal data directly onto the surgeon’s visual field, providing a “heads-up” display that enhances intuitive maneuvers and surgical safety [118].

##### Surgical Robotics

Robotic platforms offer unparalleled stability and precision for delicate tasks such as osteotomy, drilling, and prosthetic implantation. As robotic technology matures, including the integration of haptic feedback, the safety and repeatability of TMJ interventions will be significantly elevated [119].

#### 5.2.3. Tissue Engineering and Regenerative Medicine

The utilization of autologous or allogeneic stem cells—such as mesenchymal stem cells (MSCs) and induced pluripotent stem cells—for the regeneration of TMJ cartilage, the articular disc, and associated ligaments has emerged as a major research hotspot. The transplantation of stem cells encapsulated within biomaterial scaffolds and supplemented with specific growth factors can successfully induce the regeneration of functional hyaline and fibrocartilage, offering a promising alternative to conventional alloplastic prostheses and autogenous bone grafts [120].

Additionally, the application of biochemical signaling molecules and growth factors holds substantial developmental prospects. Previous investigations have demonstrated that cytokines such as transforming growth factor-beta, bone morphogenetic protein-2 (BMP-2), and insulin-like growth factor-1 (IGF-1) are instrumental in modulating cellular differentiation and tissue regeneration [121,122,123]. Future paradigms will increasingly focus on integrating localized sustained-release systems with precision regulation technologies to maximize regenerative efficiency.

## 6. Conclusions

TMJ defects arising from congenital malformations, bony ankylosis, and neoplastic pathologies pose a significant threat to a patient’s physiological function and psychosocial well-being. Consequently, the implementation of scientifically grounded and highly individualized reconstructive strategies is paramount for successful clinical rehabilitation.

At present, treatment options for TMJ reconstruction are highly developed, encompassing diverse modalities such as costochondral free grafting, vertical ramus osteotomy, and mandibular ramus distraction osteogenesis for localized or complex condylar defects. Furthermore, alloplastic total joint replacement and various vascularized bone flaps, often coupled with simultaneous orthognathic surgery for concurrent dentofacial deformities, provide versatile solutions tailored to specific age groups and clinical presentations. The selection of an optimal surgical paradigm necessitates a comprehensive evaluation of multifactorial determinants, including patient age, the biological nature of the lesion, the anatomical extent of the defect, and the severity of associated skeletal discrepancies.

Despite these advancements, the field of TMJ reconstruction still faces substantial biological and technical hurdles. Through the continued integration of multidisciplinary innovation—particularly in the realms of digital technology and regenerative medicine—future interventions are poised to become more precise, minimally invasive, and predictable. The ultimate goal remains the transition from simple structural repair to true bio-integrated functional regeneration, offering revolutionary therapeutic prospects for patients suffering from debilitating TMJ disorders.

## Figures and Tables

**Table 1:** A summary of etiological bases, TMJ phenotypes and maxillofacial features for major congenital syndromes.

Syndrome	Etiological Basis	TMJ Phenotypes & Maxillofacial Features
Hemifacial Microsomia (HFM)	Asymmetric failure of 1st and 2nd branchial arch development.	Asymmetric condylar hypoplasia/aplasia (OMENS Types I–III), absent glenoid fossa, reduced ramus height, secondary canting of the occlusal plane.
Treacher Collins Syndrome (TCS)	Autosomal dominant; mutations in TCOF1, POLR1C, or POLR1D genes.	Bilateral symmetrical condylar and mandibular hypoplasia, aplastic/hypoplastic zygomatic arches, steep mandibular angle, anterior open bite.
Cleidocranial Dysplasia (CCD)	Autosomal dominant; mutations in RUNX2 gene.	Delayed ossification of skull/clavicles, hypoplastic/deformed condyles, altered glenoid fossa morphology, supernumerary teeth, midface hypoplasia.
Nager Syndrome	Autosomal dominant; mutations in SF3B4 gene.	Severe bilateral mandibular and condylar hypoplasia, frequently associated with TMJ ankylosis, absent retrocondylar structures.

## Data Availability

No new data were created or analyzed in this study. Data sharing is not applicable to this article.
